# Paternal Exercise Improves the Metabolic Health of Offspring via Epigenetic Modulation of the Germline

**DOI:** 10.3390/ijms23010001

**Published:** 2021-12-21

**Authors:** José Maria Costa-Júnior, Sandra Mara Ferreira, Mirian Ayumi Kurauti, Diana L. Bernstein, Elena G. Ruano, Vasumathi Kameswaran, Jonathan Schug, Ricardo Freitas-Dias, Claudio C. Zoppi, Antonio C. Boschero, Camila A. M. de Oliveira, Gustavo J. Santos, Everardo M. Carneiro, Klaus H. Kaestner

**Affiliations:** 1 Department of Structural and Functional Biology, Biology Institute, State University of Campinas (Unicamp), Campinas 13083-864, SP, Brazil; josefisioexer@gmail.com (J.M.C.-J.); biosandramara@gmail.com (S.M.F.); mirian.kurauti@hotmail.com (M.A.K.); freitas-dias@hotmail.com (R.F.-D.); czoppi@unicamp.br (C.C.Z.); boschero@unicamp.br (A.C.B.); emc@unicamp.br (E.M.C.); 2 Department of Genetics and Institute for Diabetes, Obesity and Metabolism, Perelman School of Medicine, University of Pennsylvania, Philadelphia, PA 19104, USA; dianaber@mail.med.upenn.edu (D.L.B.); vask@mail.med.upenn.edu (V.K.); jschug@mail.med.upenn.edu (J.S.); Kaestner@pennmedicine.upenn.edu (K.H.K.); 3 Diabetes and Obesity Laboratory, August Pi I Sunyer Biomedical Research Institute (IDIBAPS), Campus Casanova, Casanova, 14308036 Barcelona, Spain; egonzalez@ciberdem.org; 4 Spanish Biomedical Research Centre in Diabetes and Associated Metabolic Disorders (CIBERDEM), 14308036 Barcelona, Spain; 5 Department of Biosciences, Federal University of Sao Paulo, Santos 11015-020, SP, Brazil; 6 Islet Biology and Metabolism Lab., Department of Physiological Sciences, Center for Biological Sciences, University Federal of Santa Catarina (UFSC), Florianópolis 88040-900, SC, Brazil

**Keywords:** transgenerational inheritance, DNA methylation, exercise training, molecular mechanisms, metabolism

## Abstract

Background/Aims: Epigenetic regulation is considered the main molecular mechanism underlying the developmental origin of health and disease’s (DOHAD) hypothesis. Previous studies that have investigated the role of paternal exercise on the metabolic health of the offspring did not control for the amount and intensity of the training or possible effects of adaptation to exercise and produced conflicting results regarding the benefits of parental exercise to the next generation. We employed a precisely regulated exercise regimen to study the transgenerational inheritance of improved metabolic health. Methods: We subjected male mice to a well-controlled exercise -training program to investigate the effects of paternal exercise on glucose tolerance and insulin sensitivity in their adult progeny. To investigate the molecular mechanisms of epigenetic inheritance, we determined chromatin markers in the skeletal muscle of the offspring and the paternal sperm. Results: Offspring of trained male mice exhibited improved glucose homeostasis and insulin sensitivity. Paternal exercise modulated the DNA methylation profile of PI3Kca and the imprinted H19/Igf2 locus at specific differentially methylated regions (DMRs) in the skeletal muscle of the offspring, which affected their gene expression. Remarkably, a similar DNA methylation profile at the PI3Kca, H19, and Igf2 genes was present in the progenitor sperm indicating that exercise-induced epigenetic changes that occurred during germ cell development contributed to transgenerational transmission. Conclusion: Paternal exercise might be considered as a strategy that could promote metabolic health in the offspring as the benefits can be inherited transgenerationally.

## 1. Introduction

The dramatic increase in the incidence of Type 2 diabetes over the past several decades supports the overriding influence of lifestyle changes rather than alterations in our genetic makeup on disease progression [1,2,3,4,5]. Furthermore, many individuals presenting with metabolic diseases such as obesity or diabetes benefit from changes in lifestyle such as increased physical activity and/or diminished caloric intake [2,6,7,8]. Environmental factors may affect not only the somatic cells but also the gametes, which means that metabolic states that are experienced by the parents may influence the health of the next generation [1,9,10]. An imbalance in energy intake, whether sub-nutrition or over-nutrition, in the parental generation has been linked to the development of metabolic disease in human, drosophila, and rodent offspring [11,12,13,14], which, in turn, increases the risk for the development of cancer, endocrine disruption, and other metabolic complications [3,15,16,17]. This phenomenon was observed first by Barker, who established the ‘developmental origin for health and disease’ (DOHAD) hypothesis [16,18,19]. While many studies have confirmed the developmental origins of diseases [11,17,20,21], the molecular mediators that transmit parental phenotypes to the next generation are largely unknown.

The imprinted gene modulation has been proposed as potential candidate to explain the DOHAD. These genes present monoallelic expression and, in this way, are vulnerable to the paternal or maternal modulation in the progeny [22]. The intergenomic conflict is among the theories that try to explain the physiological role of imprinted genes. In accordance with this hypothesis, the father propagates his genome in a pattern to prioritize the metabolic sources to the offspring in the detriment of the mother, whereas the maternally expressed monoallelic genes act in an opposite manner [23,24].

IGF-2 and H-19 are among the most well-described imprinted genes [25]. The IGF-2 gene is a paternally imprinted gene that encodes the protein IGF-2 that presents a high affinity with the insulin receptor and is related to mitogenesis [26] and mitochondrial biogenesis [27]. On the other hand, H-19 is a maternally imprinted gene that also is related to muscle insulin sensitivity [28]. Moreover, not only imprinted genes are involved in the epigenetic inheritance modulation. For instance, the muscle Pi3Kca loci DNA methylation is increased in the offspring from obese fathers [3,24]. Moreover, it was associated with a reduction in its gene expression and insulin sensitivity. In this way, we thought that paternal training could induce an opposite effect.

It is essential to determine if transgenerational effects of exercise on offspring phenotype can be transmitted through a purely germ-cell mediated process [29]. To this end, we investigated the effects of paternal exercise on glucose metabolism in progeny. We demonstrate that the offspring of trained male mice (*Mus musculus*) have improved glucose homeostasis and insulin sensitivity. Paternal exercise also modulated the DNA methylation profile of the insulin signaling pathway gene Pi3kca as well as the imprinted genes Igf2/H19 at specific differentially methylated regions (DMRs) in the skeletal muscle of the offspring.

Gene expression in the skeletal muscle of the progeny corresponded with the observed changes in DNA methylation, with offspring of trained fathers showing higher Pi3kca and Igf2 gene expression, while that of H19 was reduced. Remarkably, a similar DNA methylation profile at the Pi3kca, Igf2 IGF2/H19 loci was present in the progenitor sperm, indicating that exercise-induced epigenetic changes in the germline contributed to the transgenerational transmission.

## 2. Results

### 2.1. Paternal Exercise Improves Glucose Homeostasis in the Offspring

**Figure 1:**
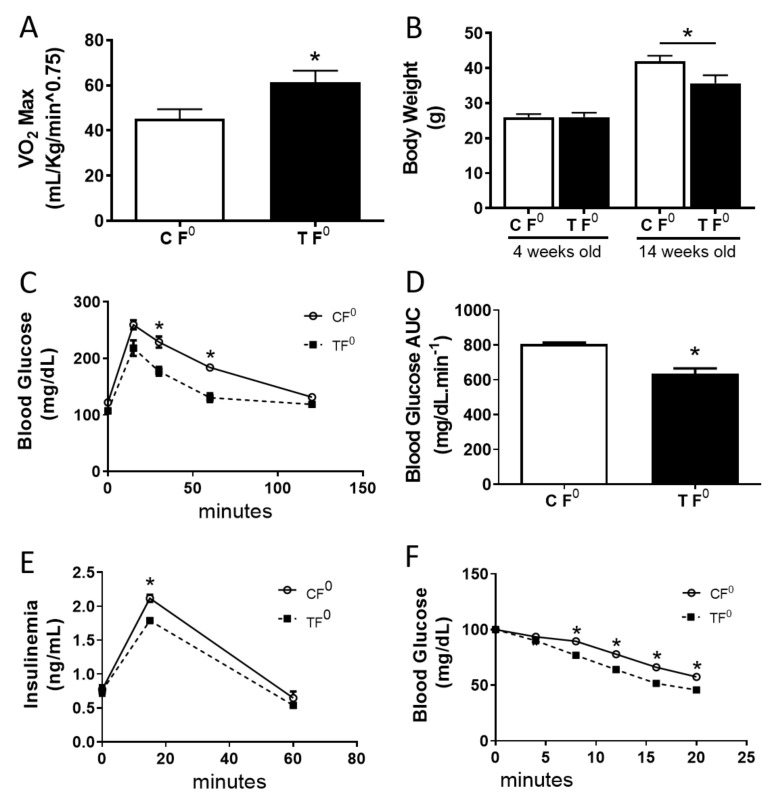
Controlled exercise regimen improves metabolic health in male mice**.** (**A**) Maximal oxygen consumption of sedentary male progenitor mice CF0 (white bar) and trained male progenitor TF0 (black bar) after the exercising training program. (**B**) Body weight of sedentary male progenitor mice CF0 (white bar) and trained male progenitor TF0 (black bar) before and after the exercise training program. (**C**) Glucose tolerance test, (1 g/Kg, b.w.) of sedentary male progenitor mice CF0 (solid line) and trained male progenitor TF0 (dashed line). (**D**) Area under the curve from glucose tolerance test of sedentary male progenitor mice CF0 (white bar) and trained male progenitor TF0 (black bar). (**E**) Plasma insulin levels during the glucose tolerance test of sedentary male progenitor mice CF0 (solid line) and trained male progenitor TF0 (dashed line). (**F**) Insulin tolerance test (1 U/Kg/b.w.) of sedentary male progenitor mice CF0 (solid line) and trained male progenitor TF0 (dashed line). Data are presented as the mean ± SEM. * *p* ≤ 0.05 vs. CF0 using two-tailed *t*-test. n = 5–6.

We first tested a novel, well-controlled paradigm of endurance training that controlled for the intensity of training and limits the effects of adaptation to exercise (details in Experimental Design). As predicted, after the endurance training period, the endurance-trained (TF0) male mice showed augmented VO2 maximum (Figure 1A), lower body weight gain (Figure 1B), improved glucose tolerance (Figure 1C,D), reduced plasma insulin levels after a glucose challenge (Figure 1E), and increased insulin sensitivity (Figure 1F) compared to the sedentary control group.

**Figure 2:**
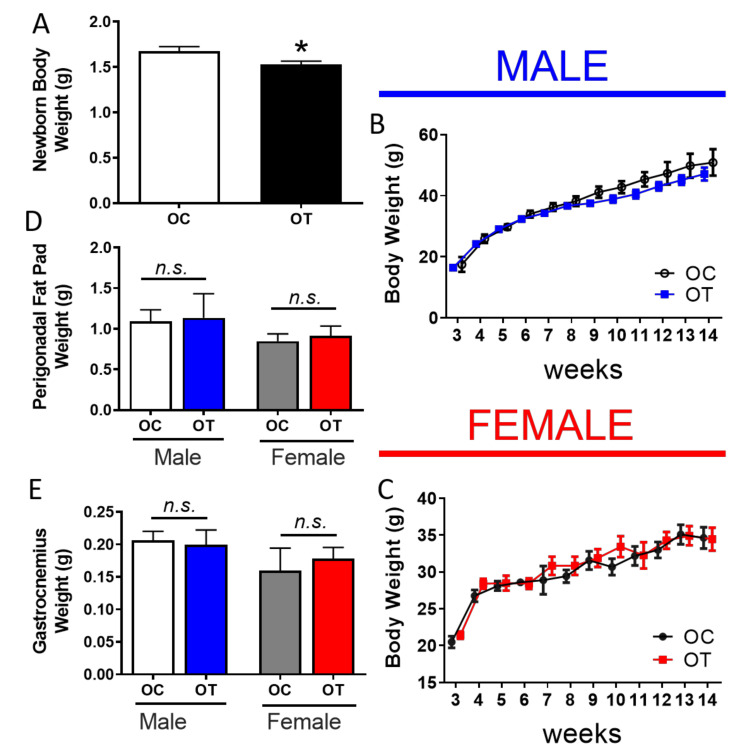
Offspring body composition.(**A**) Newborn body weight of OC mice (white bar) and OT mice (black bar). (**B**) Body weights were assessed for 12 weeks, from weaning (week 3) to euthanasia (week 14) in OC male (open circles) and OT male offspring (blue squares). (**C**) Body weights were assessed for 12 weeks, from weaning (week 3) to euthanasia (week 14) in OC female (black circles) and OT female progeny (red squares). (**D**) Perigonadal fat pad weights of OC male mice (white bar), OT male mice (blue bar), OC female mice (gray bar), and OT female mice (red bar). (**E**) Gastrocnemius weight of OC male mice (white bar), OT male mice (blue bar), OC female mice (gray bar), and OT female mice (red bar). Data are presented as the mean ± SEM. * *p* ≤ 0.05, *n.s.* = non-significant vs. OC using two-tailed *t*-test. n = 12 (panel **A**) n = 6 (panel **B**–**E**).

Both groups of males, trained or sedentary, were mated to sedentary females and the offspring were characterized using multiple physiological parameters. The bodyweight at birth was lower in the offspring that descended from the trained male progenitors compared to those that were sired by sedentary males (Figure 2A). However, between weaning and the end of our intervention (12 weeks), the body weight did not differ significantly between the two groups of offspring (Figure 2B,C). Likewise, body composition after euthanasia, measured by perigonadal fat pad (Figure 2D) and skeletal muscle weight (Figure 2E), was not affected by the exercise training of the fathers. It is possible that the absence of alterations in the body composition during the later life between the offspring groups could be attributed to the fact both OC and OT were kept in a sedentary behaviour and chow diet.

**Figure 3:**
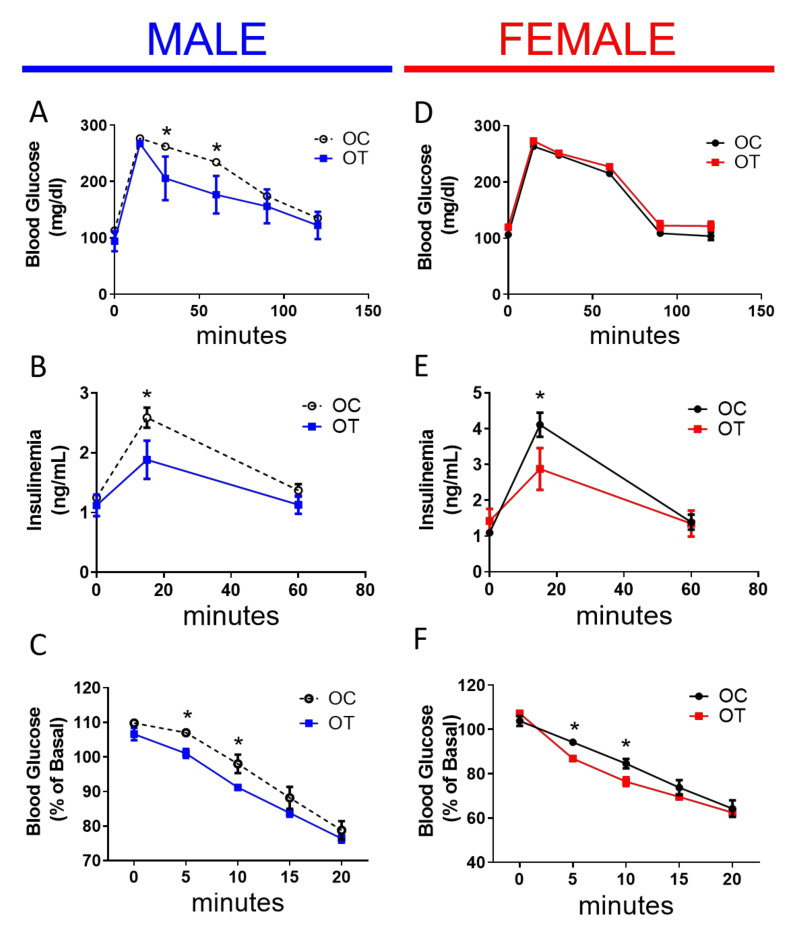
Offspring sired by trained male mice show improved metabolic health. (**A**) Intraperitoneal glucose tolerance tests (1 g/Kg, b.w.) (**B**), insulinemia during the ipGTT (**C**) insulin tolerance tests (1 U/Kg/b.w.) of male offspring sired by OC male (dashed black lines) or OT male mice (blue line). (**D**) Intraperitoneal glucose tolerance tests (1 g/Kg, b.w.) (**E**), and insulinemia during the ipGTT (**F**) insulin tolerance tests (1 U/Kg/b.w.) of female offspring sired by OC male (solid black line) or OT male mice (red lines). Data are presented as the mean ± SEM. * *p* ≤ 0.05 vs. OT using two-tailed *t* test. n = 6.

Despite the identical weight and body composition, we observed a significant improvement in the glucose tolerance in male offspring from exercise-trained fathers (OT male mice group) (Figure 3A,D), and offspring of both genders demonstrated reduced plasma insulin levels after a glucose challenge (1 g/kg/body wt) (Figure 3B,E). Thus, the offspring of male mice that were exposed to intensive exercise training displayed augmented insulin sensitivity (Figure 3C,F).

### 2.2. Paternal Exercise Alters DNA Methylation in the Skeletal Muscle of Their Offspring

**Figure 4:**
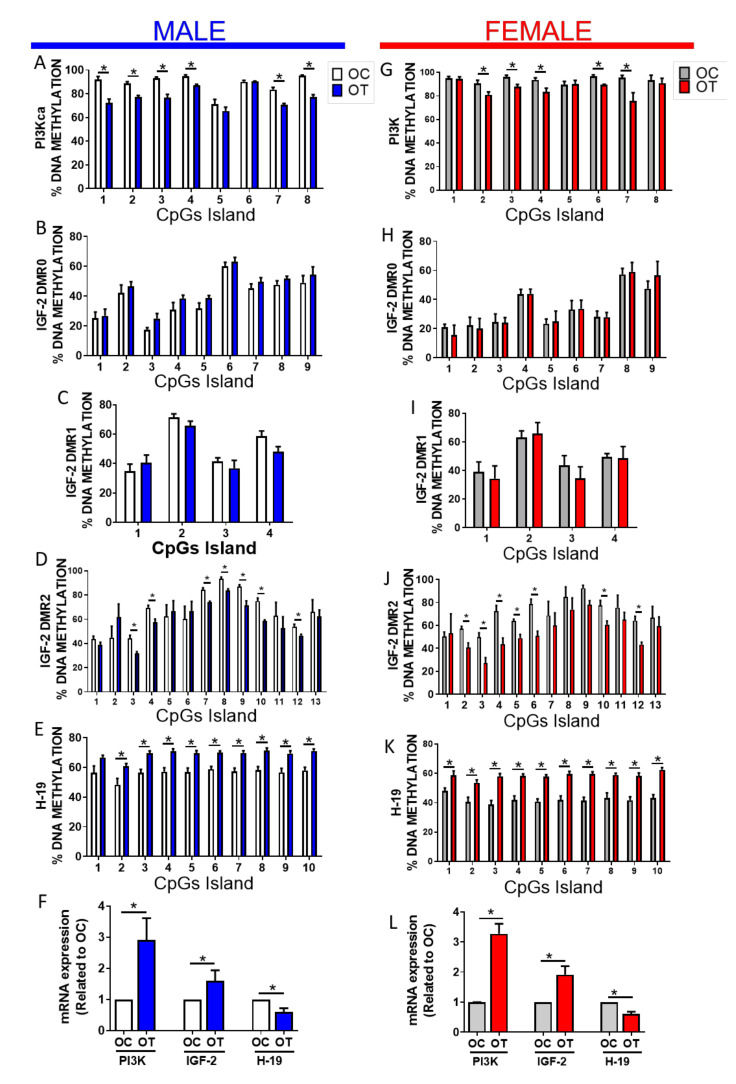
DNA methylation and gene expression analysis in the skeletal muscle offspring from sedentary or exercise-trained progenitors. (**A**–**E**) DNA methylation percentage at the (**A**) PI3Kca intragenic region of (Coordinates: chr3:32356060-32356676), (**B**) the Igf2 DMR-0; (Coordinates: chr7:149839803-149839897), (**C**) the Igf2 DMR-1 (Coordinates: chr7:149840128-149840338), (**D**) the Igf2 DMR-2; (Coordinates: chr7:149850877-149856303) and (**E**) the H19 DMR (Coordinates: chr7: 149767514-149839759) in skeletal muscle of male offspring sired by exercise-trained (OT, blue bars) or sedentary (OC, open bars) males. (**F**) Gene expression as determined by qRT-PCR of PI3Kca, Igf2 and H19 in the skeletal muscle of male offspring sired by exercise-trained (OT, blue bars) or sedentary (OC, open bars) males. (**G**–**K**) DNA methylation percentage at the (**G**) PI3Kca intragenic region of, (**H**) the Igf2 DMR-0; (**I**) the Igf2 DMR-1 (**J**) the Igf2 DMR-2; and (**K**) the H19 DMR in skeletal muscle of female offspring sired by exercise- trained (OT, red bars) or sedentary (OC, grey bars) males. (**L**) Gene expression as determined by qRT-PCR of PI3Kca, Igf2 and H19 in the skeletal muscle of female offspring sired by exercise- trained (OT, red bars) or sedentary (OC, grey bars) males. Data are presented as the mean ± SEM. * *p* ≤ 0.05 using two-tailed *t*-test. n = 6.

To determine the molecular underpinnings of the transgenerational effect of paternal exercise training on glucose homeostasis and insulin sensitivity in the offspring, we focused on DNA methylation analysis, an epigenetic marker. Targeted methylation analysis of skeletal muscle showed significant decreases in the CpG methylation at the Pi3kca locus (Figure 4A,G) and the differentially methylated region 2 (DMR2) of the Igf2 locus (Figure 4D,J) in both male and female offspring from exercised fathers. No differences in the methylation profile were seen at the Igf2 DMR0 (Figure 4B,H) and Igf2 DMR1 (Figure 4C,I). Paternal exercise also resulted in a reduction of the methylation level at the H19 DMR (Figure 4E,K). Importantly, steady-state mRNA expression in skeletal muscle of offspring correlated with altered DNA methylation levels, in that the gene expression of Igf2 and Pi3kca was increased, whereas that of H19 was decreased in the offspring that descended from trained male progenitors (Figure 4F,L). In sum, paternal exercise caused a transgenerational improvement in glucose homeostasis that correlated with epigenetic changes, particularly decreased methylation, and increased the expression of Pi3kca which encodes a key component of phosphoinositide 3 kinase, an essential activator of the insulin/AKT signaling pathway.

### 2.3. Endurance Exercise-Training Alters the DNA Methylation Profile in the Male Progenitor Sperm

**Figure 5:**
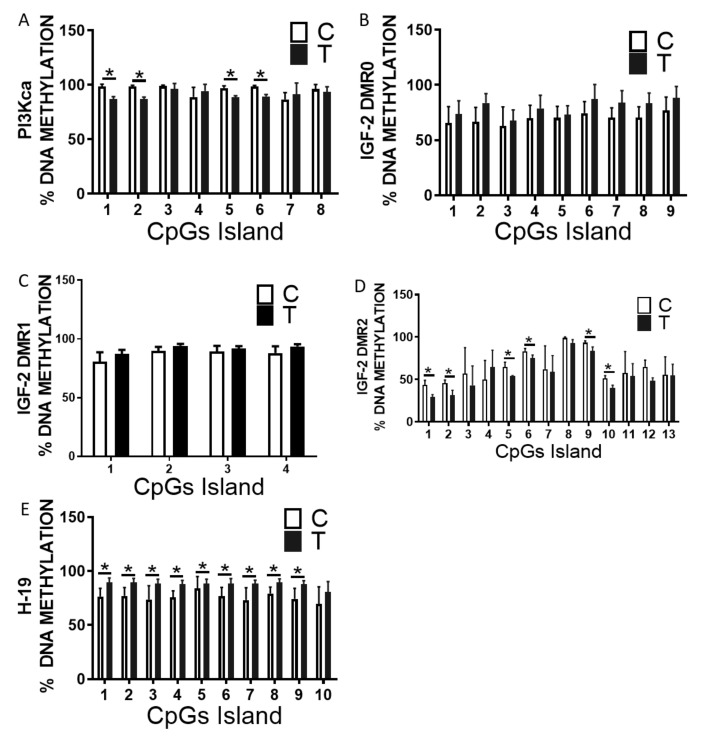
DNA methylation analysis of progenitor sperm from sedentary or exercise-trained males. DNA methylation percentage at the (**A**) PI3Kca intragenic region, (**B**) Igf2 DMR-0, (**C**) Igf2 DMR-1, (**D**) Igf2 DMR-2, and (**E**) H19 DMR in sperm of exercise-trained TF 0 male (black bars) or sedentary CF0 mice (white bars) males. Data are presented the as mean ± SEM. * *p* ≤ 0.05 using two-tailed *t*-test. n = 6.

For true transgenerational epigenetic inheritance, the epigenetic marker must be present in the germ cells of the progenitor generation. To assess whether the DNA methylation changes we discovered in the skeletal muscle of the offspring of exercise-trained fathers were indeed epigenetically inherited, we evaluated the methylation profile of the sperm of F0 males that had undergone 10 weeks of endurance exercise-training. Remarkably, similar to what we had observed in the skeletal muscle of the offspring, exercise training decreased the CpG methylation at the Pi3kca (Figure 5A) and the Igf2 DMR2 loci (Figure 5D). In addition, and again reflecting the situation in the skeletal muscle, no differences were found in the methylation profile at DMR0 (Figure 5B) and DMR1 of the Igf2 locus (Figure 5C), whereas the methylation level of the H19, DMR was decreased compared to sperm of sedentary control mice (Figure 5E).

## 3. Discussion

Barker’s hypothesis of a developmental origin of disease, proposed 26-years ago, has been confirmed by multiple studies over the past decades [1,3,11,19]. In contrast, few studies have investigated the reverse scenario where the question of whether the healthy lifestyle of the parents can lead to improved health in the offspring [29,30]. Here, we demonstrate that paternal exercise improved glucose tolerance and insulin sensitivity in the progeny. Strikingly, this was associated with hypomethylation of the Pi3kca gene, a key player in insulin signaling, in the skeletal muscle. Remarkably, the epigenetic changes that were present in the offspring reflected the altered methylation status of paternal sperm, suggesting that physical exercise can be considered an epigenetically heritable health intervention which can be transmitted via the male germline.

Previous studies had failed to demonstrate a beneficial effect of paternal training on glucose homeostasis in offspring [31,32]. In fact, prior work reported that paternal exercise induced impairment in glucose tolerance, increased plasma insulin levels, and potentiated weight gain that was induced by a high-fat diet via reduced energy expenditure in the offspring. The discrepancies between the previous studies and the work that is described here are likely due to the different exercise protocols that were employed. Both Guth et al. [33] and Murashov et al. [32] used voluntary wheel running which does not allow for control of either exercise volume or intensity, as mice have free access to the wheels throughout the study. In addition, total energy expenditure does not increase with exercise in a dose-dependent manner. Instead, there is a threshold above which an increase in exercise intensity or duration decreases energy expenditure by other organ systems in such a way that total energy expenditure plateaus [34,35]. Evolutionarily, such a constrained energy expenditure model would be advantageous.

In our experimental model, the intensity and volume of exercise were precisely controlled, resulting in a higher exercise intensity than what had been employed previously [32,33]. In fact, our exercise protocol efficiency is in agreement with others [36,37,38] who had also observed improvements in glucose tolerance and insulin sensitivity in trained mice. Likewise, forced swim training of male progenitors also induced improved glucose homeostasis in female offspring [30]. These results suggest that energy expenditure, as well as the volume and/or intensity of exercise training, are critical factors in the capacity to induce beneficial transgenerational effects via paternal exercise [38,39,40,41].

Epigenetic modifications have been considered the main molecular mechanism by which the developmental origins of health and disease occur [1,42,43]. Among them, the addition of a methyl group to the 5-carbon position of cytosine residues, also known as DNA methylation, seems to be the most important, although the exact means by which DNA methylation dynamics control transgenerational inheritance has not been defined [3,44,45,46]. A previous study reported hypermethylation at the PI3kca locus in insulin-resistant offspring, which were the descendants of mice that had been fed a high-fat diet [3]. Thus, we propose that paternal exercise training induces the opposite effect on the offspring as we observed lower methylation levels at several CpG dinucleotides at the PI3Kca locus in male and female offspring compared to offspring from sedentary fathers. This hypomethylation, in turn, correlated with higher PI3kca gene expression that was observed in the offspring, which may explain, at least in part, the improvement in their insulin sensitivity. The elevated expression of both PI3kca and Igf2, that was caused by reduced DNA methylation at their specific loci, can increase PI3k/AKT pathway activation [47,48]. Therefore, we propose that epigenetically inherited activation of the PI3k/AKT pathway contributes to the improved insulin sensitivity that is seen in offspring of exercise-trained males.

To deepen our knowledge about how paternal exercise training can promote health in sedentary offspring, we also analyzed the methylation level of DMRs at the imprinted Igf2/H19 locus in the skeletal muscle of the progeny. Imprinted genes are a class of genes that are mono-allelically expressed from either the maternally or paternally inherited chromosome in a process that is regulated by DNA methylation [46,49]. There are several theories that have been proposed to explain the existence of imprinting in mammals; here we consider the intergenomic conflict hypothesis proposed by Haing (1991) and Moore (1991).

Between IGF2/H19 genes exists a 2.4 kb long, differentially methylated region (DMR) that is required for the mono-allelic expression of both the H19 and Igf2 genes, and, therefore, is called an imprinting control region. There are two paternally methylated regions, DMR1 that functions as a silencer of the maternal allele, and DMR2 that functions as an enhancer of the paternal allele and controls IGF2 expression. A third region also exists, DMR0, which is preferentially methylated on the maternal allele and its modulation is restricted to the placenta [50]. The methylation patterns of DMR1 and DMR2 have been shown to vary according to the tissues and seem to be established during the early embryogenesis.

In line with the intergenomic conflict hypothesis, the skeletal muscle of offspring from exercised male progenitors showed a reduction in the methylation level of the paternal imprinted gene IGF2 at DMR-2, while the methylation at the maternal-controlled imprinted gene H19 locus was increased. Gene expression was correlated with the methylation status, where we observed higher IGF2 and lower H19 mRNA expression in the skeletal muscle of offspring from the trained fathers. Although our observations agree with the afore-mentioned hypothesis [51,52], specific studies with genetic manipulations or targeted epimutations will be required to elucidate how paternal physical exercise can modulate imprinted genes and to what degree these changes in imprinting contribute to the physiology of the offspring.

Paternal exercise training could affect the offspring’s metabolism via several different mechanisms. For example, paternal lifestyle changes can affect spermatogenesis and the composition of the seminal fluid [3,53,54,55]. Physical exercise also affects spermatogenesis and germ cells, increasing sperm concentration and motility [56]. The reduction of the fat pad around the gonads could also affect sperm physiology [57], and possible cross-talk between the contracting skeletal muscle and gonads via myokines must also be considered [32,58,59,60]. We focused on the premise that physical exercise can alter the epigenome in sperm and thus carry the acquired characteristics to the next generation, affecting the offspring’s phenotype. Previous studies had demonstrated that paternal exercise also could affect the sperm epigenome due to altered DNA methylation and microRNA expression [30,32].

Here, we demonstrate that increased energy expenditure of male progenitors can affect the DNA methylation status of specific genes in the skeletal muscle of their offspring, with very similar changes to the DNA methylation status seen in the paternal sperm. Our results extend previous findings which demonstrated that the alteration of DNA methylation in sperm can be correlated with the somatic cell DNA methylation profile in offspring [3,61]. However, how paternal exercise training impacts sperm DNA methylation remains an important question for future investigations.

## 4. Materials and Methods

### 4.1. Animal Care and Husbandry

The study was performed with Swiss mice that were obtained from the breeding colony at the Unicamp animal facility. The mice were maintained at 22 ± 1 °C on a 12 h light-dark cycle. At the end of the experiment, themice were fasted for 12 h, anesthetized in a CO_2_ chamber, and then killed by decapitation. We divided 30-day-old male progenitors into two groups: a control group that was kept sedentary (CF0) and a trained group (TF0), both fed with a standard rodent chow. The exercise was performed in the light part of the day cycle and its protocol consisted of treadmill running, 1 h per day, 5 days per week for 10 weeks, at an intensity of 70–80% of maximal oxygen consumption.

### 4.2. Maximal Oxygen Consumption (VO2Max)

VO2Max was assessed during the pre-training period, and after every two weeks of training in a sealed treadmill at a 25° incline that was coupled to a gas analyzer (Oxylet system, Pan Lab/Harvard Instruments, Spain) to allow for training intensity adjustments. The oxygen uptake data were recorded using the Metabolism software (Pan Lab/Harvard Instruments, Spain). VO2Max was assumed when the oxygen uptake reached a plateau despite increasing treadmill speed. After an eight minute warm-up, the test started at a speed of 15 cm.sec-1. The treadmill speed was increased by 15 cm.sec-1 every 45 sec until mice were unable to maintain the elicited effort level [62].

### 4.3. Mating Scheme

During the last week of the exercise protocol (10th week), we placed 10 control and 10 trained male mice singly into a cage with one virgin female mouse each, keeping one male and one female mouse per cage with free access to chow and water in the light part of the day cycle (from 18:00 to 08:00) for five consecutive days. Each day, the males were returned to their respective cages at 08:00 and kept there until 18:00. The male mice of the TF0 group were maintained on the exercise protocol as before. To avoid a bias from detraining, the exercise section occurred at 08:00 h and the trained mice were returned to the female cage at 18:00. The female mice were fed a standard chow during the entire period (mating, gestation, and lactation) and were kept sedentary. At weaning, both male and female offspring were distributed into two groups, as described below. To avoid any breastfeeding bias, we included in the study only litters with 8–10 pups. To compose the experimental groups we selected one male and one female mouse randomly from each mating pair and divided the offspring into four groups: 6 male and 6 female offspring from the sedentary control fathers (OC), and 6 male and 6 female offspring from the trained fathers (OT). All of the groups were fed a standard chow diet from weaning to 90 days. The offspring body weight was determined weekly from birth to euthanasia. The F0 males were killed, and the sperm were collected after the last day (5th day) of the matting scheme.

### 4.4. Intraperitoneal Glucose Tolerance Test

For the intraperitoneal glucose tolerance test (ipGTT), food was withdrawn 12 h before the experiment. A total of 24-h after the last exercise session (F0) or 90-days p.p. (F1 male and female), the mice were weighed and a basal blood sample was taken from the tip of the tail (t0). The glucose concentration was measured with an Accu-Check Advantage II glucometer (Roche). Subsequently, the mice received an intraperitoneal glucose solution load (1 g/kg body wt) and additional blood samples were collected and glucose levels were determined at 15, 30, 60, 90, and 120 min after the glucose injection. The area under the curve for the blood glucose was calculated by the trapezoidal method [63]. Blood that was collected at 0, 15, and 60 min was also used for plasma insulin analysis by radioimmunoassay.

### 4.5. Intraperitoneal Insulin Tolerance Test

For the intraperitoneal insulin tolerance test (ipITT), food was withdrawn 4 h before the test. A total of 24-h after the last exercise session (F0) or 90-days p.p. (F1 male and female), the mice were injected with 1 U/Kg bodyweight of human insulin (Biohulin R, Biobras, Brazil). Blood samples were collected and glucose was measured (Accu-Check Advantage II Glucometer, Roche) before (t0) and 4, 8, 12, 16, and 20 min after insulin administration.

### 4.6. DNA Methylation Analysis

Genomic DNA (gDNA) from sperm and skeletal muscle (soleus) was extracted using the Qiagen Allprep DNA/RNA mini kit (cat.#80204) following the manufacturer’s instructions. A total of 500 ng of gDNA was treated with sodium bisulfite to convert unmethylated cytosines to uracils using the Qiagen Epitech Bisulfite Kit (cat.#59104) per the manufacturer’s protocol. DNA methylation analysis was performed using BisPCR2, as described previously [44].

Bisulfite-converted genomic DNA was PCR amplified to enrich for regions of interest for analysis. The primers that were directed to target regions were modified with the following partial adapter overhangs: PCR#1 Left Primer Overhang: 5′- ACACTCTTTCCCTACACGACGCTCTTCCGATCT-3′; PCR#1 Right Primer Over-hang: 5′-GTGACTGGAGTTCAGACGTGTGCTCTTCCGATCT-3′. The primers that were directed to the intragenic regions of PI3Kca (F: GAAAGAGTATAGTGAGGAGAAATAAGA; R: AACAACAAAACTACCATAACTATTTTC; Coordinates: chr3:32356060-32356676), H19 Differentially Methylated Regions (DMRs) (F: GGTTTTTTAAGTGATTTTTTGGGTAGG; R: ATTACAACTAATTAAATTTTCTCCCCTATC; Coordinates: chr7: 149767514-149839759), IGF2 DMR-0 (F: ATGTTTGTGGGAGTGGTATAGGTA; R: CCCATCTCAACAACTATCCT; Coordinates: chr7:149839803-149839897), IGF2 DMR-1 (F: ACTCCCAACAAATTTACAAATCTC; R: TACCTAACAAAATCCAAAAATTCATCTCC; Coordinates: chr7:149840128-149840338) and IGF2 DMR-2 (F: GAGTGGAGTAGAGAGATTTTAGTG; R: ACCCCAATAAACACTAACTAAAATTATCT; Coordinates: chr7:149850877-149856303), were designed using the Qiagen Pyromark assay Design software. The PCR reactions were prepared with the Qiagen PyroMark PCR Kit (cat.#978703) per the manufacturer’s recommendations using 2.8 ng of bisulfite-converted gDNA template per reaction and pooled in approximately equimolar ratios and purified with the Qiagen QIAquick PCR Purification kit (Cat.#28104) per manufacturer’s instructions. Barcoding of the PCR product pools from step one was performed as described by Bernstein et al. [44], and the final sample concentrations were determined using the Qubit fluorometer dsDNA high sensitivity assay (Life Technologies, Carlsbad, CA, EUA). The molarity of the libraries was quantified using the KAPA library quantification assay (Roche, Basel, Switzerland, Cat.# KK4873) before next-generation sequencing. The list of the primers used in this work is in the Supplementary Materials.

### 4.7. Next-Generation Sequencing

Next-generation sequencing was carried out on an Illumina MiSeq using a Reagent Kit v2 following the manufacturer’s instructions. Briefly, a 2 nM pool of BisPCR2 libraries and 2 nM PhiX control were each denatured for 5 min with 0.2 N NaOH and diluted to final concentrations of 6 and 8 pM, respectively. The denatured pool was spiked with 10% denatured PhiX control and 600 μL of the prepared sample was loaded into the reagent cartridge. Sequences were aligned to the in silico bisulfite-converted mouse genome using the BS Seeker program [64] and any CpGs that was already covered by the first sequencing read were ignored in the second sequencing read in paired-end sequencing.

### 4.8. Quantitative Real-Time PCR

Total RNA was extracted using the Qiagen Allprep DNA/RNA mini kit (cat.#80204). A total of three micrograms of total RNA were reverse-transcribed using SuperScript II Reverse Transcriptase (Life Technologies) to synthesize cDNA. Quantitative RT-PCR was performed on a Stratagene Mx3000P using 2× Brilliant III SYBR Green qPCR Master Mix plus ROX reference dye (Agilent Technologies). The thermal profiles were set according to the manufacturer’s protocol. The mRNA levels were normalized to Hprt1: (Forward primer: CCTAAGATGAGCGCAAGTTGAA, reverse: CCACAGGACTAGAACACCTGCTAA), PI3Kca: (Forward primer: CCACGACCATCTTCGGGTG, reverse: ACGGAGGCATTCTAAAGTCACTA) H19: (Forward primer: GGAATGTTGAAGGACTGAGGG, reverse: GTAACCGGGATGAATGTCTGG), Igf2: (Forward primer: TCAGTTTGTCTGTTCGGACC, reverse: CACTCTTCCACGATGCCAC).

### 4.9. Statistics

The results are expressed as the mean ± SEM. The data were analyzed by unpaired two-tailed *t*-tests using Statistica 12 software. Significance was set at *p* < 0.05. Before unpaired two-tailed *t*-test analysis, homoscedasticity and a Gaussian distribution of the samples were assumed based on the Levene Test and the Kolmogorov-Smirnov normality test. We adopted an effect size ≥ of 0.50 and a study power of 0.50.

## 5. Conclusions

In summary, we demonstrated that, just as the developmental origins of disease could be related to the paternal environment, improved metabolic health of offspring can also be stimulated by paternal lifestyle changes. Our data suggest that the modulation of a progenitor’s energy expenditure and exercise regimen can be as important as energy intake to trigger alterations in the germ cells epigenome and, consequently, influence glucose homeostasis in the offspring.

## Data Availability

The data that support the findings of this study are available from the corresponding author upon reasonable request.

## Supplementary Materials

The following are available online at https://www.mdpi.com/article/10.3390/ijms23010001/s1.
