# Supplementary figures and images for: Paternal Exercise Improves the Metabolic Health of Offspring via Epigenetic Modulation of the Germline
Source: Int J Mol Sci. 2021 Dec 21;23(1):1. doi: 10.3390/ijms23010001 (PMC8744992; doi:10.3390/ijms23010001)

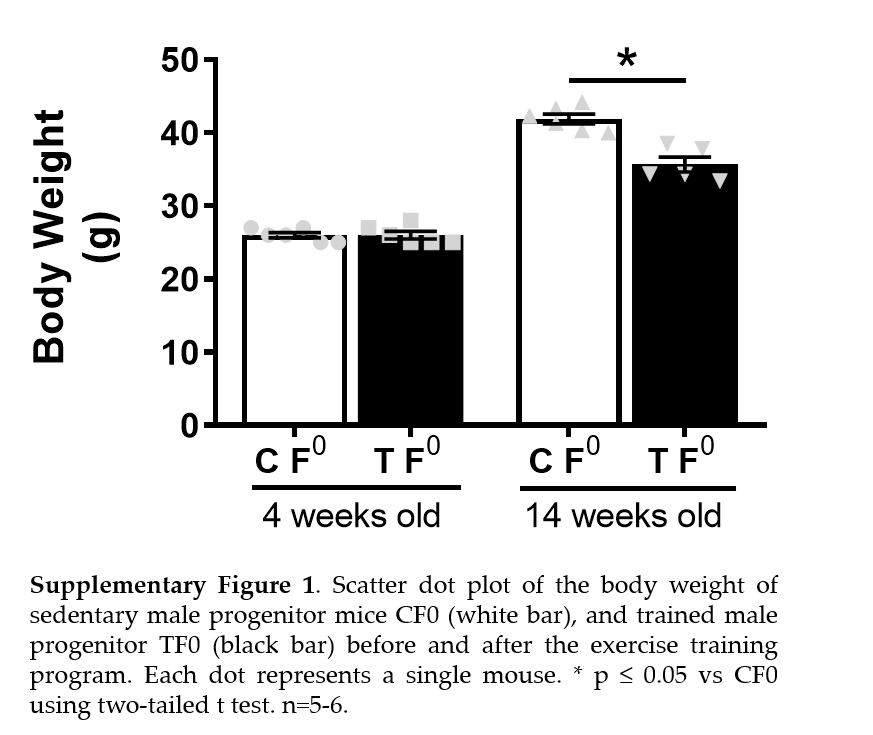

Supplement: Supplementary file 1 [file ijms-23-00001-s001.zip › Supplementary Figure S1.tif]

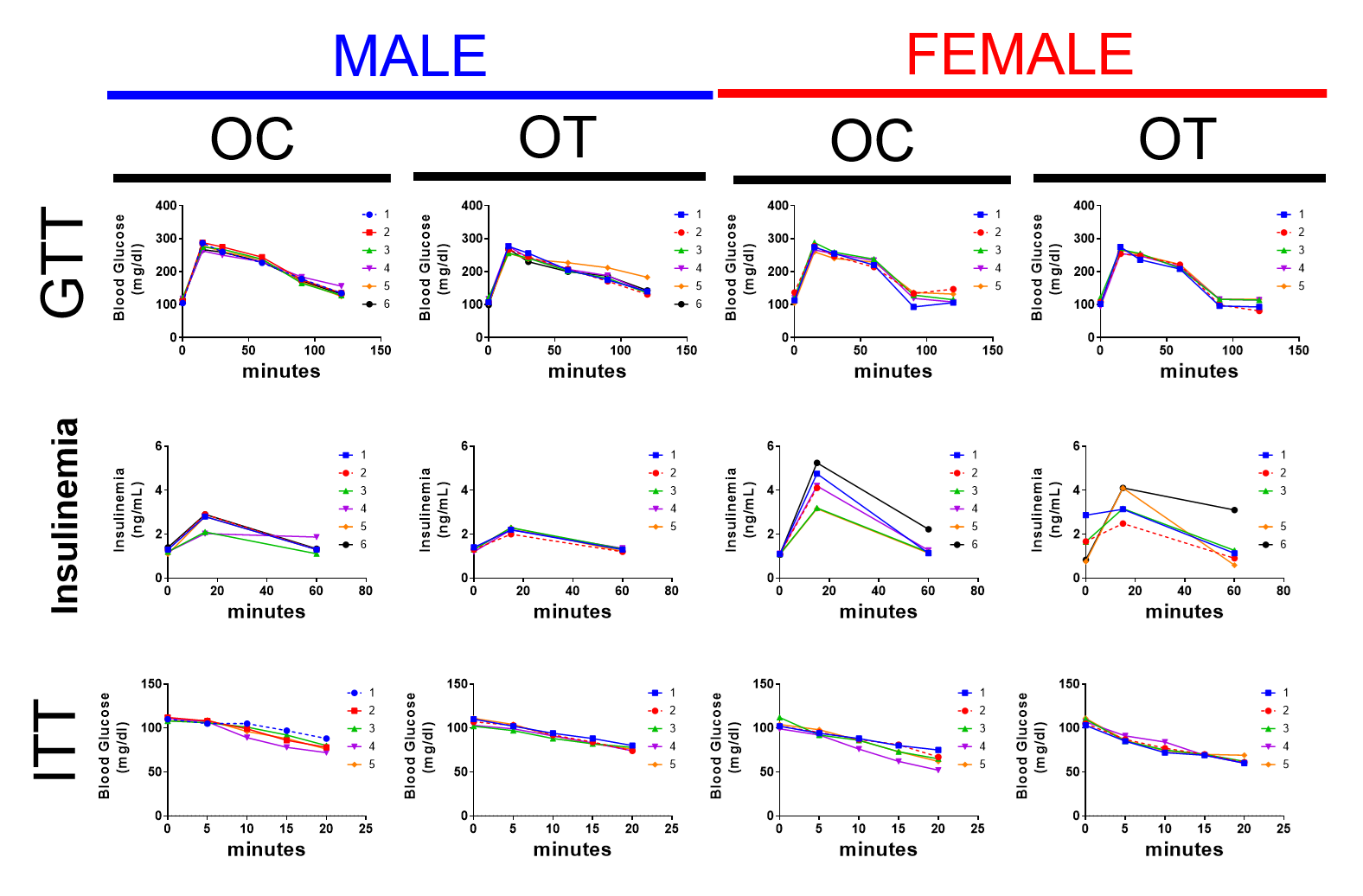

Supplement: Supplementary file 1 [file ijms-23-00001-s001.zip › Supplementary Figure S2.tif]

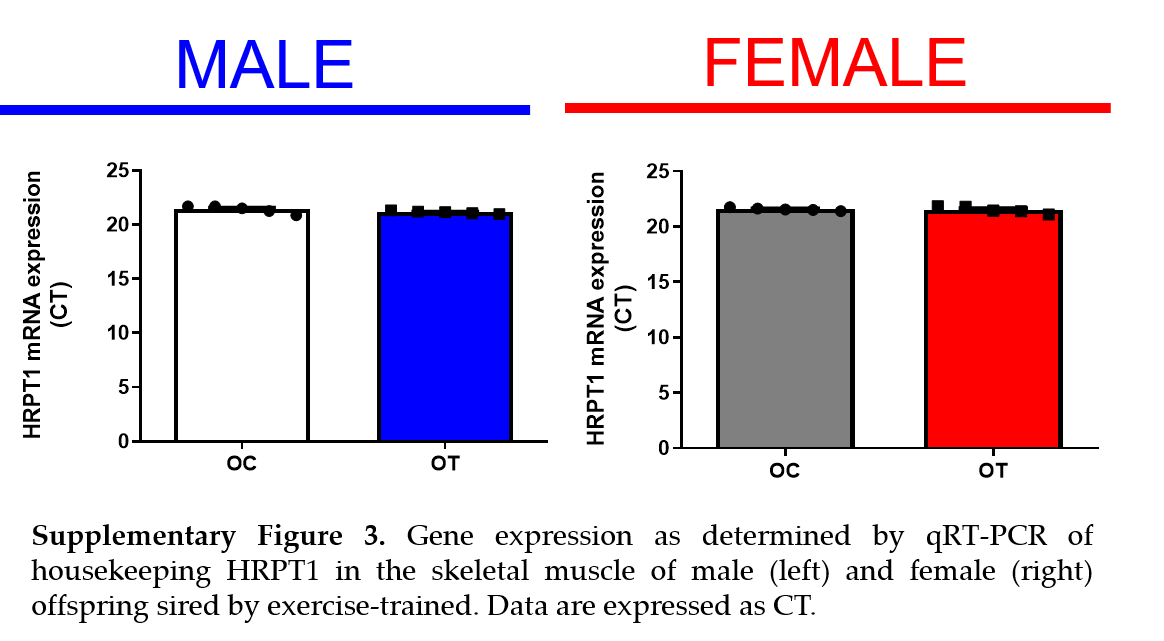

Supplement: Supplementary file 1 [file ijms-23-00001-s001.zip › Supplementary Figure S3.tif]

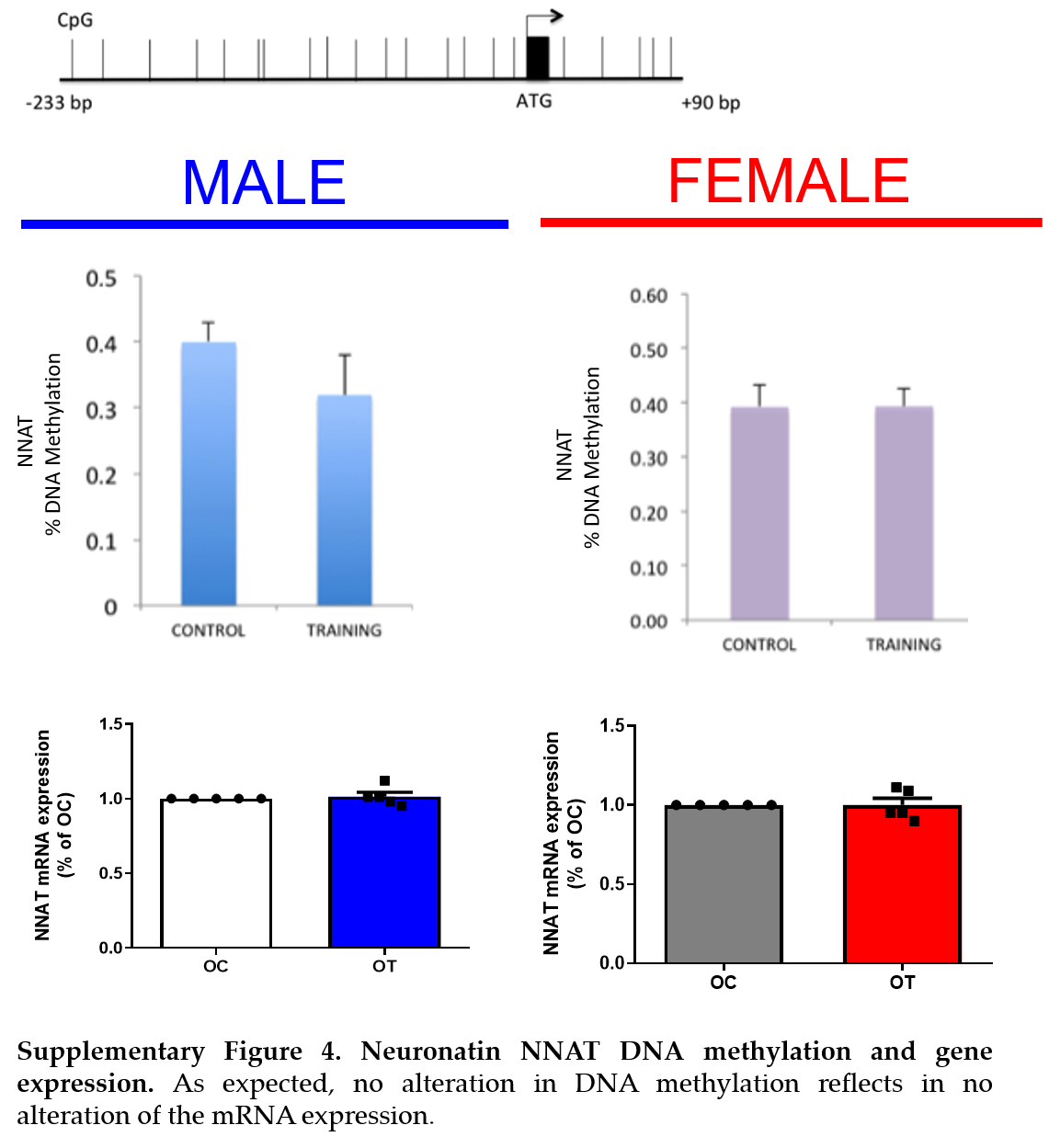

Supplement: Supplementary file 1 [file ijms-23-00001-s001.zip › Supplementary Figure S4.tif]
